# Atelocollagen-Embedded Chondrocyte Precursors as a Treatment for Grade-4 Cartilage Defects of the Femoral Condyle: A Case Series with up to 9-Year Follow-Up

**DOI:** 10.3390/biom11070942

**Published:** 2021-06-25

**Authors:** Hwa-Chang Liu, Tzu-Shang Thomas Liu, Yen-Liang Liu, Jyh-Horng Wang, Chih-Hung Chang, Tiffany Ting-Fang Shih, Feng-Huei Lin

**Affiliations:** 1 Department of Orthopaedic Surgery, Taiwan Adventist Hospital, Taipei 10556, Taiwan; hcliu@ntuh.gov.tw; 2 Department of Orthopaedic Surgery, National Taiwan University Hospital, Taipei 10063, Taiwan; jhwang@ntuh.gov.tw; 3 Southern California Bone and Joint Clinic, Apple Valley, CA 92307, USA; oakleyzeros@gmail.com; 4 Master Program for Biomedical Engineering, China Medical University, Taichung 40402, Taiwan; allen.liu@cmu.edu.tw; 5 Department of Orthopaedic Surgery, Far Eastern Memorial Hospital, New Taipei 22060, Taiwan; orthocch@mail.femh.org.tw; 6 Department of Radiology, National Taiwan University Hospital, Taipei 10063, Taiwan; ttfshih@ntu.edu.tw; 7 Department of Biomedical Engineering, National Taiwan University, Taipei 10617, Taiwan

**Keywords:** osteoarthritis, osteonecrosis, cartilage regeneration, chondrocyte precursor, mesenchymal stem cell

## Abstract

We demonstrated the safety and efficacy of autologous chondrocyte precursor (CP) cell therapy in repairing Grade 4 cartilage defects of medial femoral condyles. The autologous bone marrow mesenchymal stem cells of each participant were isolated, amplified, and then differentiated into CPs in atelocollagen. Neotissues made of CPs were implanted into cartilage defects with an average cell density of 4.9 ± 2.1 × 10^6^ cells/cm^2^ through arthrotomy. The knee function was evaluated with the International Knee Documentation Committee (IKDC) subjective knee form. Patients’ knee functions significantly improved by the 28th week (IKDC score = 68.3 ± 12.1), relative to the initial functionality before the CP therapy (IKDC score = 46.1 ± 16.4, *p*-value = 0.0014). Nine of these twelve patients maintained good knee functions for 9 years post-implantation (IKDC score = 69.8 ± 12.3) at levels higher than the pre-implantation values (*p*-value = 0.0018). Patients were evaluated with MRI and arthroscopy, and the defective sites exhibited a smooth surface without a gap between the implant and host tissue. This study demonstrates that autologous CPs successfully engraft into the host tissue and result in the re-formation of hyaline-like cartilage, thereby improving the impaired knee functions. Most importantly, no adverse event was reported during this long-term follow-up period.

## 1. Introduction

Arthritis is an epidemic group of joint disorders and a leading source of chronic pain and disability in the world [1]. By 2040, the number of American adults with diagnosed arthritis is projected to be 78.4 million (25.9% of all the adults) [2] due to increases in life expectancy and body mass index. The most common type of arthritis is knee osteoarthritis (OA), which accounts for >70% of the total arthritis burden [1]. If left untreated, the “wear and tear” of knee articular cartilage usually progresses into OA because cartilage is an avascular tissue with a limited capacity of self-repair. In 1743, Hunter described the impossibility of cartilage repair, stating that “once the cartilage is destroyed, it never recovers” [3,4]. Multiple surgical treatments have been developed to promote cartilage healing, such as abrasion arthroplasty [5], bone marrow stimulation through microfracture [6,7], and osteochondral autografts [8] or allografts (mosaicplasty) [9]. However, these treatments have limitations. Microfracture appears to be effective in small lesions, but it is usually associated with fibrocartilage production [10,11]. Mosaicplasty may provide a more rapid improvement than microfracture, but it is limited by donor-site morbidity for autografts [12], technical difficulty in leveling the graft at the desired position [13], and poor integration of the implant into host tissue [14,15].

In 1994, the autologous chondrocyte implantation (ACI) was introduced for the first time for the treatment of cartilage defects in the knee [16]. In the past two decades, ACI has demonstrated its efficacy for knee OA. In 2010, a systematic review concluded that, relative to microfracture and mosaicplasty, ACI may be the best option for large defects in active young patients who have had the symptoms for a short period and have not undergone a chondral surgery before [17]. Although many modifications on the ACI technique have been proposed or even completed in clinical studies (reviewed by Kyriacos Athanasiou [18]), some limitations still remain, such as the limited source of chondrocytes, donor site morbidity, low robustness of the procedure in hyaline cartilage generation, and questionable longevity of the implant or derivative tissue [19]. Thus, to surpass these limitations, various stem-cell-based methods have been developed using mesenchymal stem cells (MSCs) derived from bone marrow [20], adipose tissue [21], synovium [22], peripheral blood [23], or periosteum [24]. Because MSCs are multipotent, growth factors or biophysical/biomechanical stimuli have been used to induce the chondrogenic differentiation of MSCs or to improve the functional properties of the derived neo-cartilage tissues, including mature matrix formation [25,26]. During the chondrogenic differentiation of bone marrow MSCs toward mature chondrocytes, we have identified a unique population of chondrocyte precursors (CPs) that can secrete glycosaminoglycan (GAG) without forming lacunae, which are usually present in mature cartilage tissue.

Atelocollagen is a low-immunogenic derivative of collagen obtained by the removal of N- and C-terminal telopeptide components [27]. Due to its immunogenic and biofunctional advantages over other biomaterials, atelocollagen has been successfully employed in tissue engineering for the regenerations of cartilage [28], intervertebral disc [29], cornea [30], periodontal tissues [31], and skin [32]. In terms of cartilage tissue engineering, atelocollagen gel permitted a gradual proliferation and matrix synthesis of chondrocytes and maintained its phenotype for up to 4 weeks in vitro [28]. Sakai’s study further demonstrated atelocollagen gel served as an important carrier of MSCs for proliferation, matrix synthesis, and differentiation [29]. The Adachi group recently reported the efficacy of repairing osteochondral defects with minced cartilage embedded in atelocollagen gel [33].

The goal of this study was to evaluate the safety and efficacy of using these CPs in the repair of Grade 4 cartilage defects, as defined by the International Cartilage Repair Society (ICRS), in the weight-bearing area of medial femoral condyles.

## 2. Results

### 2.1. Demography

Twelve patients were enrolled for this study between February 2008 and April 2010 (Table 1). This study was approved by the institutional review board of the National Taiwan University Hospital (NTUH REC No.: 32MD03) and the Department of Health, Executive Yuan Taiwan (Wei-Shu-Yi No.: 0960216294), and written informed consents were obtained from all the patients. The inclusion and exclusion criteria are detailed in Materials and Methods. One patient died from a cause unrelated to the study one year after implantation, another one was lost due to emigration two years after implantation, and one patient underwent TKA on the operated knee 5.5 years after implantation.

### 2.2. CP Derivation

**Figure 1:**
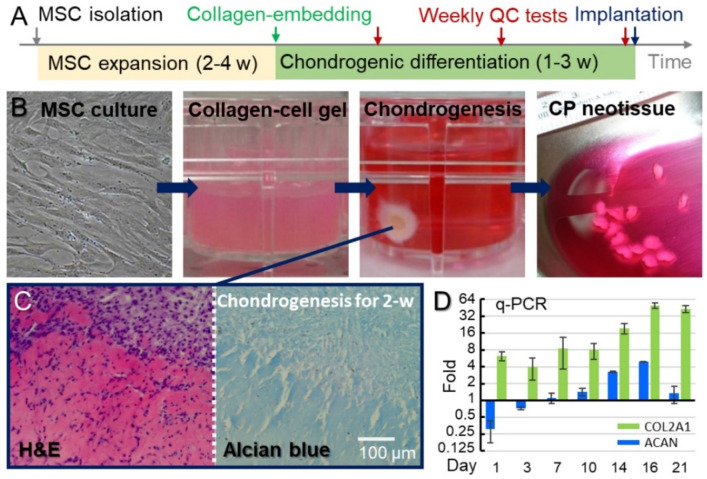
Expansion of MSCs and chondrogenic induction to CPs. (**A**) Generation of the neotissues of CPs: the bone marrow-derived MSCs were expanded through in vitro culture in a GTP lab for 2–4 weeks to obtain enough cells for CP implantation. The MSCs embedded in atelocollagen were induced to undergo chondrogenic differentiation to generate the CPs. During the differentiation, weekly quality control tests, including histological analyses, qPCR, and evaluation for bacterial, endotoxin, and mycoplasma contaminations were conducted to monitor the maturation and safety of cartilage neotissues. The qualified neotissues were then implanted into the patients. (**B**) Images of cells at different stages during the generation of the neotissues of CPs. (**C**) Histological images of neotissues of CPs stained with H&E and Alcian blue demonstrates the abundance of CPs and high GAG levels in the neotissues. (**D**) The mRNAs of two representative biomarkers of hyaline cartilage, COL2A1, and ACAN, were quantified using qPCR. The expression levels were normalized to those of undifferentiated MSCs.

Neotissues made of CPs are advantageous for de novo cartilage generation because of their in vitro preparation, which allows us to derive numerous CPs from a minute number of MSCs harvested from the patient’s own bone marrow. The bone marrow-derived MSCs collected from patients were expanded ex vivo for 2–4 weeks to obtain the sufficient cells (4.9 ± 2.1 × 10^6^ cells/cm^2^, mean ± SD) needed for the chondrogenic differentiation. Subsequently, the MSCs were embedded in atelocollagen gel and induced to undergo chondrogenic differentiation (Figure 1). The chondrogenic induction causes the contraction of the atelocollagen gel and formation of disk-shaped cartilage neotissues from MSCs (Figure 1B). As shown in Figure 1C,D, the neotissues of CPs demonstrated the histological and biomolecular features of immature chondrocytes. The chondrogenic neotissues generated from MSCs by our method and the conventional method [34] were compared with each other through histological assessment and gene expression profiling (please see the Supplementary Method S1, Figures S1 and S2 for detailed information).

### 2.3. Patient Characteristics before and after CP Therapy

**Figure 2:**
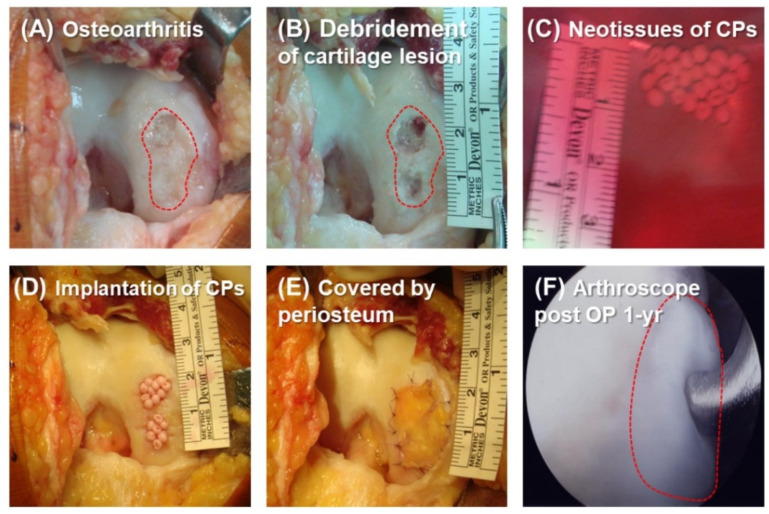
Procedure of the implantation of neotissues of CPs. (**A**) Mid-vastus arthrotomy was performed to reach the knee cartilage lesion where the damaged cartilage was debrided by abrasion arthroplasty (**B**). (**C**)The neotissues of CPs were implanted into the defects (**D**) and covered by periosteum (**E**) acquired from the proximal tibia. (**F**) Seven cases received arthroscope examinations to assess the recovery of their cartilage at least one year after the CPs were implanted.

The knee lesions were confirmed by arthroscopy. Mid-vastus arthrotomy was performed to reach the lesion in the knee cartilage, and the damaged cartilage was then debrided by abrasion arthroplasty. Representative images of the operation procedure are shown in Figure 2. The CPs were implanted at the defective sites and then covered with the periosteum. The average size of the defected areas was 1.9 ± 0.9 cm^2^ (mean ± SD, range of 0.4–3.1 cm^2^). The average number of implanted CPs was 8.8 ± 5.1 × 10^6^ (range of 2.7–20.6 × 10^6^) (Table 1). After implantation, the knee was immobilized at 30 degrees flexion for three days, and then the free motion of the knee and partial weight-bearing were allowed for two weeks. Afterward, full weight-bearing was initiated. The unoperated contralateral knee was used as the control.

**Figure 3:**
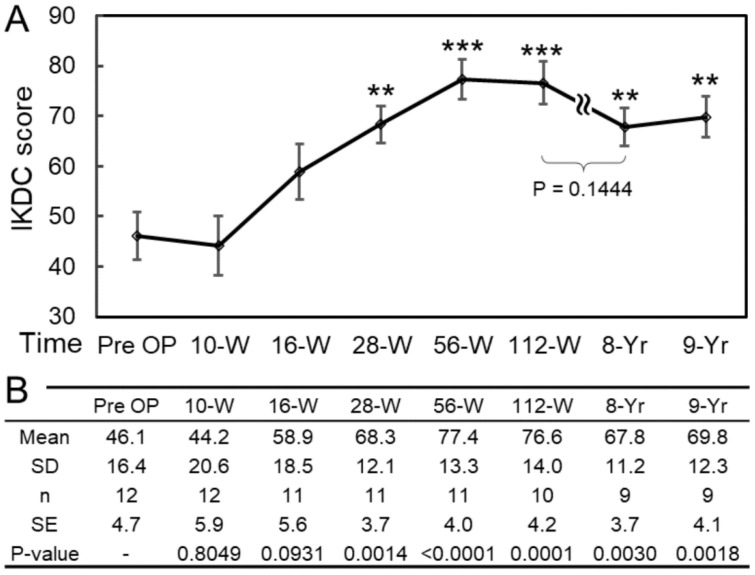
Assessment of knee functions during daily activities by IKDC scores. (**A**) The knee functions of the patients were significantly improved 28 weeks after the CP implantation. After a 9-year follow-up, the mean IKDC score was still approximately 69.8, which is significantly higher than the pre-operation value. The unpaired *t*-test was conducted to determine the difference between the means of the IKDC scores before and after the CP implantation at different time points. In addition, there was no significant difference between the 2-year and 8-year groups, indicating that the improvement in the knee function is sustainable. The error bars stand for standard errors. (**B**) The table shows detailed information about the IKDC scores and the *p*-values by comparing each group with the pre-OP. SD: standard deviation; n: number of cases; SE: standard error. The statistical comparison was performed using unpaired *t*-test, where the asterisk represents the statistical significance: *** *p <* 0.001, ** *p <* 0.01. The error bar represents the standard error of the mean.

We applied the International Knee Documentation Committee (IKDC) subjective knee evaluation criteria to evaluate the knee function before and after the CP therapy. Before the implantation, the average IKDC score of the 12 patients was 46.1 ± 16.4 (mean ± SD). The average IKDC scores at 56 and 112 weeks after the implantation were 77.4 ± 13.3 (Case 6 lost) and 76.6 ± 14.0 (Cases 4 and 6 lost), respectively. The average IKDC score of 9 patients (Case 4, 6, and 10 lost) at 9 years after the implantation was 69.8 ± 12.3, which was still significantly higher than the pre-implantation average (Figure 3).

**Figure 4:**
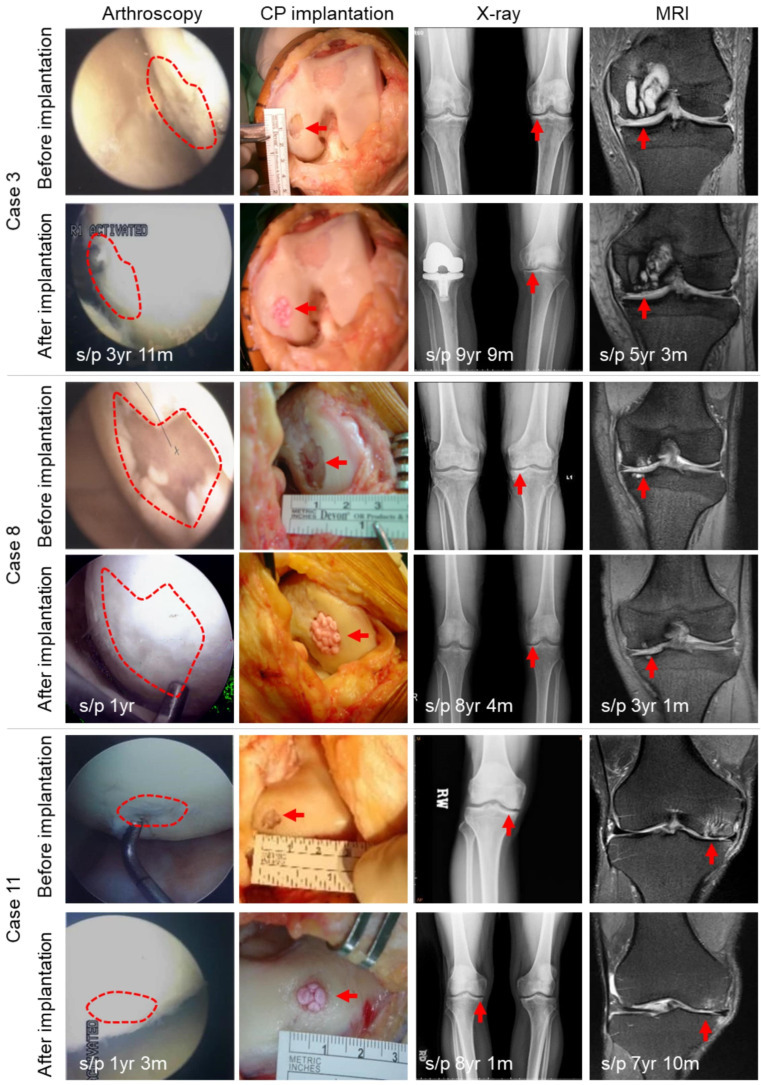
Assessments of the cartilage lesion and recovery before and after the CP implantation. Cartilage lesion as seen via arthroscopy, knee arthrotomy, X-ray, and MRI before and after the CP implantation for three representative patients (Cases 3, 8, and 11). The implanted CP tissue was softer than the native cartilage tissue on palpation with a probe. The defect sites are circled by the red dashed lines in the arthroscopy images and indicated by the red arrows on the other three types of images.

There were no infections, inflammations, adhesions, loose body, or tumor formations in the CP-implanted knee, as examined by arthroscopy, X-ray, and MRI (representative images are shown in Figure 4, and more arthroscopic images in Figure S3). The Kellgren–Lawrence (K–L) grading system was used for classifying the severity of OA based on x-rays. The CP-implanted knees also showed no substantial changes in K–L grade (*p*-value = 0.34) as blindly evaluated by three orthopaedic surgeons, whereas untreated contralateral knees developed severer OA (Figure S4). Using weight-bearing radiography, we measured the femoral-tibial angles (FTAs) based on the anterior-posterior views of the knees. Before implantation, the 12 knees to be operated on had FTAs of 178.9 ± 3.3 degrees (178–184 degrees). The final FTAs of the 9 patients who were followed up for 9 years after implantation became 180.1 ± 3.7 degrees (175–186 degrees). Although there was no statistically significant improvement in the varus deformity of the knees, there was also no evidence that the deformity worsened in the 9-year follow-up. Three of the nine untreated contralateral knees had to undergo total knee arthroplasty (TKA) during the follow-up period.

The magnetic resonance observations of cartilage repair tissue (MOCART) system was used to evaluate the imaging features of CP implants, and the results are summarized in Table 2. In total, 11 patients underwent MRI before the implantation and one year afterward. The defects in 7 of them (63.6%) were completely filled, 2 patients had hypertrophy, and 2 patients had incomplete filling. A total of 8 patients (72.7%) showed complete graft integration into the border zone, one had a visible demarcating border, and 2 patients still had visible defects. Intact surface tissue appeared in 8 patients (72.7%), whereas 3 patients still had a damaged surface. The structure of the repaired tissue was homogenous in 9 patients (81.8%). Isointense signal was seen in 8 patients (72.7%); 3 patients had a moderately hyperintense signal, but no marked hyperintense signal was observed. As far as the repair of the subchondral bone was concerned, 6 patients (54.5%) had an intact subchondral lamina, and all the patients had non-intact subchondral bone. Finally, no patients exhibited adhesion, and 9 patients (81.8%) showed no evident effusion.

### 2.4. Histologic Analysis of Repaired Tissue

**Figure 5:**
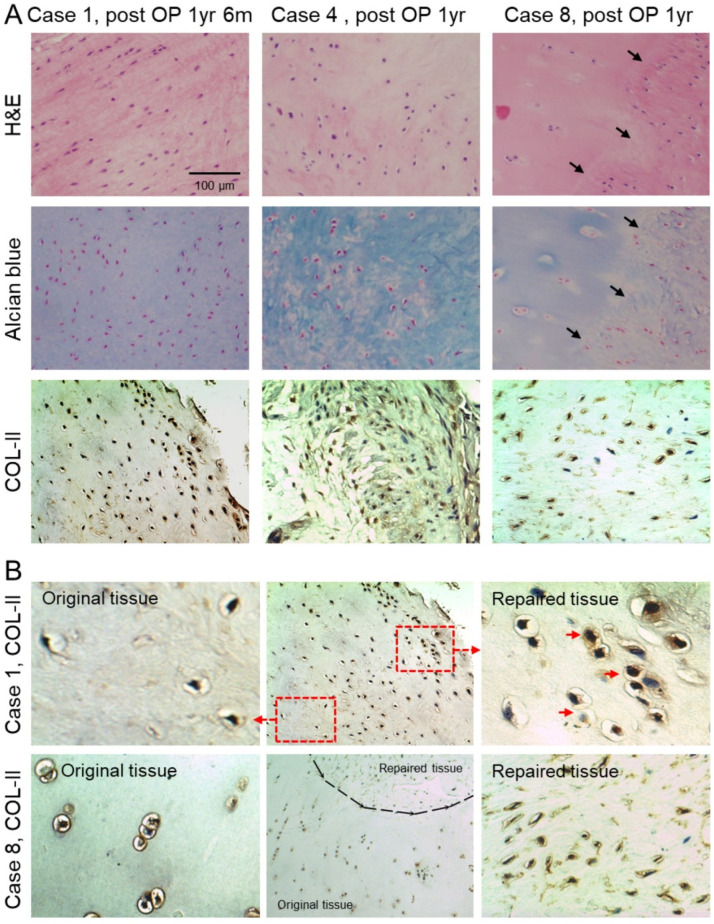
Histological analyses of biopsy specimens collected by the arthroscope. (**A**) Histological images of the biopsy specimens of three representative subjects (Cases 1, 4, and 8). The H&E staining, Alcian blue staining, and immunohistochemistry were performed to visualize the tissue morphology and expression levels of GAG/Collagen II (COL-II), respectively. The black arrows point out the integration of the implanted neotissues. (**B**) The immunohistochemical analysis for COL-II in the implanted cartilage tissue from Cases 1 and 8 demonstrate the integration of the implanted CPs into the original cartilage at the recipient sites. Compared with the original cartilage tissue, the CPs were relatively smaller in size and denser in number, and some of them showed lacuna development (red arrows) 1.5 years after the CP implantation.

In total, 7 patients consented to a second-look arthroscopy and biopsy 1–8 years after the CP implantation (Table 1). We observed that the de novo cartilage tissue was significantly softer than the original cartilage (Figure 4), and the histology images showed the formation of lacuna-like chondrocytes in the de novo cartilage tissue (Figure 5). GAG and collagen type II were observed in the biopsy specimens, and 3 specimens of 3 patients are presented in Figure 5. It was noted that the CPs in the de novo tissue were smaller and denser than the cells in the original cartilage tissue (Figure 5B).

The CPs obtained by our culture method and the chondrogenic cells derived from MSCs by the conventional methods [34] (Figure S1) were compared with each other through assessment of their histological features (Figure S2) and transcriptomic profiles (Figure S5). The transcriptomic analysis included 8 genes related to cartilage development: *COL1A1*, *COL2A1*, *COL10A1*, *ACAN*, *MMP3*, *MMP13*, *RUNX2*, and *SOX9*. We hypothesized that gradual increases in the levels of the genes related to the cartilage development would be beneficial for both cartilage maturation and integration of the implanted neotissues into the host cartilage at the implantation site (Figure S5). Please see the Supplementary Information for a detailed discussion.

## 3. Discussion

Recently, a systematic review has indicated that there are limited high-quality evidence and long-term follow-up observations for stem cell therapy in osteoarthritis, although MSC therapy has been shown to have a positive effect on OA patients [35]. In this case series study, we demonstrated the safety and efficacy of CP therapy for cartilage defects from osteoarthritis or osteonecrosis based on several clinical assessments, including arthroscopy, IKDC score, MRI, and histology. During the 9-year follow-up, no adverse reactions, such as infection, effusion, adhesion, loose body, or tumor formation, was noted, unlike the observation that implanted periosteum causes adverse reactions [36].

Sufficient cell seeding density in the cartilage defects would be critical for the recovery. Although there are no clinical studies that have specifically investigated the effect of cell seeding density on the clinical outcome, many clinicians continue to use 0.5–12 × 10^6^ chondrocytes/cm^2^ in ACI [37], which almost approximates the chondrocyte densities found in native adult articular cartilage (2.4 × 10^6^ cells/cm^2^ in the superficial layer) [38] and has been associated with favorable clinical outcomes [37]. In terms of MSC-based cell therapy, Fini’s group reviewed 11 case series and reports that used autologous bone marrow-MSCs for cartilage repair with the cell seeding densities ranging from 0.8 to 7.1 × 10^6^ MSCs/cm^2^, and they concluded that larger cartilage defects can be repaired by a two-step technique involving in vitro expansion of MSCs, rather than by the one-step strategy [39], most probably because of the higher cell numbers achieved by the in vitro expansion procedure. In this study, the number of implanted CPs ranged from 2.7 to 8.6 × 10^6^ cells/cm^2^, and these cell densities, which were comparable to those used by Fini’s group, also caused a significant improvement in knee function. In summary, the neotissue of CPs is advantageous for cartilage repair because of the in vitro preparation, which allows generation of numerous chondrogenic cells from a minute number of MSCs harvested from the patient himself/herself.

Besides the cell density used in the implantation, the implantation efficiency, which means the number of cells surviving the implantation process, is critical for the repair of the defect. Relative to the scaffold-free ACI, CPs are embedded in atelocollagen, and the neotissues of CPs are cell-leak-free and easy to implant for any shape of cartilage defect (Figure 2). Most importantly, our method can achieve more uniform cell distribution in the recipient site. These advantages may contribute to the efficacy and durability of CP therapy.

Before the operation, the native knees had a mean FTA of 178.9 ± 3.3 degrees. Therefore, they were varus knees if a normal FTA is defined as 173–175 degrees. However, no additional valgus osteotomy was performed to correct the varus alignment in this study. Even so, sustained joint spaces at the medial compartment of the knee (Figure 4) and good MRI results (Figure 4) support the fact that CPs repaired the cartilage defects effectively, such that no increase in varus deformity of the knee was detected in the 9-year follow-up. In addition, subjects still maintained satisfactory knee functions, with IKDC scores of 69.8 ± 12.3 by the 9th year after the operation. The conditions slightly deteriorated as the follow-up progressed; however, this outcome might be due to aging. The soft CP tissue noted in arthroscopy might not have a clinical drawback on the knee because the patients still had good IKDC scores (Figure 3). The soft implanted tissue might become more mature and harder with time, as Mazor et al. have reported [40].

The ability of engrafted cells to integrate into the recipient site and participate in the repair process is crucial for successful clinical outcomes. Here, we demonstrated that, unlike the previously reported studies using mature articular chondrocytes for cartilage repair [41], CPs exhibit efficient integration capacity. Under arthroscopic examination, the integration between the graft and the recipient site was found to be complete (Figure 4 and Figure S3). The histological analyses of the biopsy specimens also demonstrated the integration of the implanted tissue into the surrounding articular cartilage (Figure 5). The accumulation of GAG and collagen type II has been confirmed in the biopsy specimens (Figure 5A). The identified CPs, a sort of immature chondrocytes, are smaller in size, denser in population, and are usually without any lacuna compared with mature chondrocytes (Figure 5B). In vitro maturation of scaffold-free cartilage has been extensively characterized before [42]. The ECM formation starts with the production of collagen VI in the first week, followed by the production of GAG (2nd week) and then collagen II (4th week), in line with our results shown in Figure S6. At the late stage of cartilage maturation, collagen cross-linking further increases the mechanical strength of the cartilage [43]. However, the cross-linked collagen may deter the integration of the implanted neotissue into the native cartilage.

Details on the fates of implanted cells have not been presented in the clinical trials before [18] because few follow-ups include tissue biopsy. In this study, seven patients received a second-look arthroscopy and biopsy at least one year after the implantation. While the histology images showed the formation of lacuna-like chondrocytes in the implanted cartilage (Figure 5B), the neo-tissues of CPs rarely exhibited lacuna-like structure in culture (Figure 1C). The in vivo maturation of the neotissues of CPs might reflect the concept of the functional adaptation of articular cartilage. [44] The mechanical loading of the musculoskeletal system influences the properties of articular cartilage and the development of its characteristics [45]. There are clinical studies on patients showing reduced thickness (>10%) of articular cartilage in the knee joint in the absence of normal joint loading [46]. Animal models have demonstrated that the development of regional biochemical heterogeneity of cartilage is under the influence of physical loading [47], and mechanical forces are needed to maintain the normal state and functions of articular cartilage and subchondral bone [44]. The mechanical stimuli not only have both anabolic and catabolic effects on chondrocytes [48,49] but also induce the chondrogenic differentiation of MSCs [50,51]. Unlike the approaches of generating mature cartilage neotissue in vitro, the neotissues of CPs can continue chondrogenesis under the physiological loading without sacrificing the integration capability.

CP therapy demonstrated advantages on the sufficient cell density to support cartilage repair, proper integration of the implanted neotissues into the native cartilage, and well-regulated formation of hyaluronic cartilage. Although mosaicplasty is a single-stage approach, it generates donor-site morbidity and has a limitation in the size of defects. There is also a concern regarding the integration of implanted cylindrical plugs into the recipient site cartilage [13], and a smooth surface in the recipient site is needed to prevent its mirror site from cartilage damage [13]. Whereas cell therapies with in vitro cell expansion lift the limitation in defect size, ACI still suffers from the limited source of chondrocytes and donor-site morbidity [19], and MSC-based therapy has the uncertainty of the implanted tissue becoming hyaline cartilage [52].

Advanced biomaterial-based approaches for cartilage repair, such as microfabricated 3D scaffold [53], injectable stem cell-laden hydrogels [54], and 3D printed biofunctionalized scaffolds [55], have been proposed since we started this study in 2008. These new technologies can fine tune the biochemical and biomechanical properties of tissue-engineered constructs to facilitate proliferation, guide differentiation, and repair cartilage defect. Therefore, we envision that the integration of CPs with advanced tissue-engineering technology could further improve the therapeutic efficacy and the sustainability of cartilage repair.

The strength of this study is that this is one of the few clinical trials within the English literature that included a second-look arthroscopy after implantation of stem cells for articular defects. To our knowledge, only two such trials have involved biopsy of the implanted tissue [56,57]. Furthermore, only one of those two trials used autologous stem cells, and while it utilized adipose-derived MSCs with microfracture as part of its protocol, our study used bone marrow-derived MSCs and did not damage the subchondral plate. Finally, this study had a high percentage of patients followed up for >8 years and is continuing to demonstrate the efficacy of the procedure, whereas the other studies only had a maximum follow-up of 30 months.

There are limitations to the current study. First, the study is a small case series with heterogeneous patients who were diagnosed as either osteoarthritis or osteonecrosis, with ages ranging from 48 to 83 years, and the cartilage defect sizes ranged from 0.4 cm^2^ to 3.1 cm^2^. Arthroscopy was also not performed on every patient, and this was limited by patient consent. Additionally, no indentation test was performed on the implanted CPs to measure the absolute hardness, although the softer implanted part might have a clinical advantage. Finally, a biopsy was performed only on the superficial part of the implanted CPs. Nonetheless, the histologic assessments of the implanted tissues, a multitude of methods and second-look arthroscopies to clinically assess the implants, and long-term follow-up with matched controls for each knee all provided strong evidence for the long-term potential of the material by autologous CPs.

## 4. Materials and Methods

Ethical Approval: This study was approved by the institutional review board of the National Taiwan University Hospital (NTUH REC No.: 32MD03) and the Department of Health, Executive Yuan Taiwan (Wei-Shu-Yi No.: 0960216294), and written informed consents were obtained from all the patients.

Patients: Twelve patients were enrolled for this study between February 2008 and April 2010 (Table 1). The inclusion criteria were as follows: (1) chondral cartilage defects within the weight-bearing zone of the medial femoral condyle, (2) cartilage defects between 0.3 cm^2^ and 4 cm^2^, (3) patient age between 20 and 85 years, and (4) written informed consent to participate in the study. Patients were excluded if they had (1) immature skeletons, (2) severe degenerative arthritis that required TKA, (3) a history of a cruciate ligament or meniscus injury, (4) rheumatoid arthritis, (5) been under a corticosteroid treatment for more than two weeks, (6) alcoholism, (7) a history of viral infection (such as HIV or Hepatitis B), and (8) other conditions that would affect independent locomotion.

Manufacture of CPs in atelocollagen: To collect bone marrow MSCs, 10 mL of heparinized bone marrow blood was aspirated from the ilium, and then the sample was immediately delivered to a good-tissue-practice (GTP) laboratory for the generation of CPs, including steps for the isolation, expansion, and chondrogenic differentiation of the MSCs. The MSCs were isolated using the density-gradient medium Ficoll (Cat. No. 17-5446-52, GE Healthcare, Little Chalfont, United Kingdom). They were then amplified in Dulbecco’s modified Eagle’s medium with low glucose (DMEM-LG, Cat. No. 31600, Gibco, Carlsbad, CA, USA) and 10% fetal bovine serum at 37 °C in a humid environment with 5% CO_2_/95% atmosphere. The medium was changed twice a week. The cells were trypsinized and then subcultured into 3 plates every week. To generate CPs, the MSCs were harvested and embedded in Porcogen^TM^ atelocollagen solution (3% type-I/type-III collagen, Sunmax Biotechnology, Tainan, Taiwan) containing serum-free DMEM-LG only. The cells were resuspended in 0.5 mL neutralized atelocollagen solution with a final cell density of 2.6 × 10^6^ cells/mL, seeded in 24-well plates, and briefly incubated at 37 °C for the gelatinization of atelocollagen to take place. Subsequently, 2 mL/well of the following chondrogenic medium was added: serum-free medium (DMEM-LG, Cat. No. 31600, Gibco, Carlsbad, CA, USA) with ITS+ Premix (Cat. No. 354352, Corning, NY, USA), 50 µg/mL L-ascorbic acid-2-phosphate (Cat. No. 49752, Sigma–Aldrich, St Louis, MO, USA), 10^−7^ M dexamethasone (Cat. No. D4902, Sigma–Aldrich, Hamburg, Germany), and freshly added 10 ng/mL transforming growth factor beta-1 (TGF-β1, Cat. No. 100-21, PeproTech, Rocky Hill, NJ, USA). The cells were cultured in the chondrogenic medium for 7–21 days with medium changes every three days. To monitor the chondrogenic differentiation, the cell/atelocollagen mixtures were histologically analyzed and assessed for cell-specific markers by quantitative polymerase chain reaction (qPCR) 7, 14, and 21 days after the induction of the chondrogenic differentiation. Once the CPs had formed but were not fully differentiated into chondrocytes, they were assessed for bacterial, endotoxin, and mycoplasma contamination before being delivered to the hospital for implantation. The procedure for generating the neotissues of CPs is shown in Figure 1.

Surgical operation: The knee lesions were confirmed by arthroscopy. We aimed to implant 2 × 10^6^ cells/cm^2^ in the cartilage defect site. The area of the defect was estimated through the arthroscope before the collection of bone marrow MSCs. Two major factors would affect the final cell seeding densities for this study: the various proliferation capability of MSCs from individuals and the discrepancy between estimated and actual sizes of the cartilage defect. Mid-vastus arthrotomy was performed to reach the lesion in the knee cartilage, and the damaged cartilage was then debrided by abrasion arthroplasty. After implantation, the knee was immobilized at 30 degrees flexion for three days, and then the free motion of the knee and partial weight-bearing were allowed for two weeks. Afterward, full weight-bearing was initiated. The unoperated contralateral knee was used as the control.

Clinical and biomedical imaging assessments: X-ray, MRI, International Knee Documentation Committee (IKDC) scoring system [58], and arthroscopy were used for periodic evaluation of the cartilage repair. Radiographic images were evaluated according to the K–L grading scale system [59] by three doctors who were blinded as to which side was the implanted knee. Using weight-bearing radiography, we measured the FTAs based on the anterior-posterior views of the knees [60]. MRI images were taken one year after CP implantation and evaluated according to the criteria reported by Marlovits [61]. Arthroscopy, biopsy, and histological examination were carried out only with each patient’s consent. Follow-up biopsies and histological analyses were conducted to assess for the presence of GAG and collagen type II and to examine the cell morphologies in the implanted tissues. The gross appearances of the implanted tissues were scored using the ICRS cartilage repair assessment system [62].

Histological analyses of biopsy specimens and neotissues of CPs: For histological examination, the cartilage neotissues and biopsy specimens were fixed in 10% neutral buffered formalin. The samples were then dehydrated in a graded series of ethanol (70% → 80% → 95% → 100%) and embedded in paraffin wax. Consecutive 5-μm sections were cut from the paraffin blocks onto slides, deparaffinized, and stained with hematoxylin and eosin (H&E, Cat. No. 3008-1&3204-2, Muto, Tokyo, Japan) to assess the general structure under an optical microscope (Figure 1C). Alcian blue staining (Muto, Tokyo, Japan) was used to assess the amount of GAG accumulation in the implanted tissues. (Figure 1C). The immunohistochemistry was applied in assessing the protein expression of collagen type II. An anti-collagen type II antibody (Cat. No. Ab34712, Abcam, Cambridge, UK) and VECTASTAIN ABC kit (goat anti-rabbit IgG, Cat. No. PK-4001, Vector Laboratories, Burlingame, CA, USA) were used for the immunohistochemistry. In brief, the 5-μm slides of formalin-fixed paraffin-embedded blocks were deparaffinized and hydrated through xylenes and graded alcohol series. A Vector Antigen Unmasking Solution (Cat. No. H-330, Vector Laboratories, Burlingame, CA, USA) was used for antigen unmasking, and the endogenous peroxidase activity was quenched by BLOXALL blocking solution (Cat. No. SP-6000, Vector Laboratories, Burlingame, CA, USA). The sections were incubated with normal blocking serum and then the diluted primary antibody (1:100 dilution). The biotinylated secondary antibody, the ABC reagent, and then the peroxidase substrate solution (ImmPACT^®^ DAB Substrate, Cat. No. SK-4105, Vector Laboratories, Burlingame, CA, USA) were followed sequentially.

Gene expression analysis using real-time PCR: Total RNA was prepared with the RNeasy Micro Kit (Cat. No. 74004, Qiagen, Hilden, Germany), and reverse transcription was conducted with random primers by using the SuperScript first-strand synthesis system (Cat. No. 18091050, Invitrogen, Carlsbad, CA, USA) according to the manufacturer’s instructions. The cDNA was used for real-time PCR using the SYBR-Green Master PCR mix (Cat. No. 4309155, Applied Biosystems, Foster City, CA, USA) in triplicate. PCR and data collection were performed on the PRISMTM7900 Sequence Detection System (Applied Biosystems). All the gene expression results were normalized to the expression of the endogenous control GAPDH in each group, and the transcriptomic profile of each group was compared with that of the undifferentiated MSCs, which were used as a negative control. The primers were designed by Primer Express 3.0 (Applied Biosystems). These sequences are listed in Supplementary Table S2.

Statistical analysis: Pre- and post-implantation IKDC scores and FTAs were compared using unpaired *t*-tests because of the unequal sample sizes due to the missing patients during the follow-up. The equality of variances was tested by F-test, and the variances of pre- and post-implantation were equal. The statistical significances of the unpaired *t*-tests were set as *p <* 0.05, *p <* 0.01, and *p <* 0.001 for two-tailed *t*-test. Comparisons of the K–L grades were carried out using the Chi-square test, and a significance level of 0.05 was chosen. The mean, SD, and range values for all the groups were obtained.

## 5. Conclusions

The implantation of autologous CPs was found to be a promising method for effective repair of Grade 4 cartilage defects in femoral condyles, with the effect lasting for 9 years after the CP-implantation for the vast majority of the patients. The durability could be attributable to the capability of CPs in achieving both intrinsic tissue maturity and suitable integration between engrafted neotissues and recipient cartilage tissues. Most importantly, there were no infections, inflammation, adhesion, loose body, nor tumor formation in the CPs-implanted knees. The CP-based grafting technique, besides MSC and ACI, appears to be a viable long-term option for condylar lesions and may delay or prevent the need for arthroplasty.

## Figures and Tables

**Table 1:** Demography of the subjects.

Case	Gender	Diagnosis	Age on CPI	Defect Size (cm^2^) ^a^	No. Implanted CPs (×10^6^) ^b^	Date of CPI	Receive Arthroscopy	ICRS Score by Arthroscopy ^c^ PreOP/PostOP	Last Date of MRI	Last Date of X-ray
**1**	F	ON	68	2.2	6.7	20080215	20090821 20110701	1/- 1/12	20120905	20131217
**2**	M	OA	83	1.5	4.0	20080530	-	1/-	20140706	20150806
**3**	M	OA	78	1.6	9.9	20080609	20120502	3/11	20130901	20180302
**4**	F	OA	48	1.2	8.6	20080730	20090710	2/12	20101111	20101102
**5**	M	OA	62	2.4	13.7	20080808	20160725	2/6	20120924	20160718
**6**	F	OA	70	3.0	13.2	20080829	-	1/-	-	20090108
**7**	F	OA	69	3.1	8.5	20081001	-	1/-	20150830	20160801
**8**	M	ON	60	2.4	20.6	20081031	20091119	1/11	20111128	20170216
**9**	F	OA	64	1.3	6.3	20081022	-	1/-	20160421	20160225
**10**	F	OA	67	0.9	2.7	20081219	20100208	2/12	20150319	20161228
**11**	M	ON	63	0.4	3.2	20100210	20110521	2/11	20171220	20180316
**12**	M	ON	65	3.0	8.0	20100401	-	3/-	20160606	20171215

One patient (Case 10) received TKA five and a half years after the CP-implantation. Cases 2, 3, and 9 received TKA on the non-implanted knee 7, 4, and 3 years after the CP therapy, respectively. M: male; F: female; OA: osteoarthritis; ON: osteonecrosis; CPI: chondrocyte precursor implantation. The date format is year-month-day. ^a^ Average defect size: 1.9 ± 0.9 cm^2^ (mean ± SD) ranged from 0.4 cm^2^ to 3.1 cm^2^. ^b^ Averaged number of inoculated CPs: 4.9 ± 2.1 × 10^6^ cells/cm^2^ (mean ± SD) ranged from 2.7 to 8.6 × 10^6^ cells/cm^2^. ^c^ Arthroscopic evaluation of cartilage repartee is based on the arthroscopic score of ICRS cartilage repair assessment.

**Table 2:** MRI assessments of the cartilage repairs one year after the CP implantation.

**1. Degree of defect repair and filling of the defect**		**4. Structure of the repair tissue**	
Complete	7 (63.6%)	Homogenous	9 (81.8%)
Hypertrophy	2 (18.2%)	Inhomogeneous or cleft formation	2 (18.2%)
Incomplete		**5. Signal intensity of the repair tissue**	
>50% of the adjacent cartilage	1 (9.1%)	Isointense	8 (72.7%)
<50% of the adjacent cartilage	1 (9.1%)	Moderately hyperintense	3 (27.3%)
Subchondral bone exposed	0 (0%)	Markedly hyperintense	0 (0%)
**2. Integration to border zone**		**6. Subchondral lamina**	
Complete	8 (72.7%)	Intact	6 (54.5%)
Incomplete		Not intact	5 (45.5%)
Demarcating border visible (split-like)	1 (9.1%)	**7. Subchondral bone**	
Defect visible		Intact	0 (0%)
<50% of the length of the repair tissue	1 (9.1%)	Non-intact	11 (100%)
>50% of the length of the repair tissue	1 (9.1%)	**8. Adhesions**	
**3. Surface of the repair tissue**		No	11 (100%)
Surface intact	8(72.7%)	Yes	0 (0%)
Surface damaged		**9. Effusion**	
<50% of repair tissue depth	2 (18.2%)	No	9 (81.8 %)
>50% of repair tissue depth or total degeneration	1 (9.1%)	Yes	2 (18.2%)

## Data Availability

Not applicable.

## Supplementary Materials

The following are available online at https://www.mdpi.com/article/10.3390/biom11070942/s1, Method S1: Comparison of chondrogenic differentiation protocols; Table S1: Recipes of chondrogenic media; Table S2: Primer sequences for chondrogenic genes; Figure S1: Procedures of chondrogenic induction; Figure S2: Histological analysis of chondrogenic-induced MSCs; Figure S3: Arthroscopy examination before and after CP implantation; Figure S4: Grading of knee osteoarthritis by Kellgren and Lawrence system; Figure S5: q-PCR gene analysis of MSCs during chondrogenic differentiation; Figure S6: Histological analysis of extracellular matrix of the chondrogenic MSCs.
