# Supplementary material for: Atelocollagen-Embedded Chondrocyte Precursors as a Treatment for Grade-4 Cartilage Defects of the Femoral Condyle: A Case Series with up to 9-Year Follow-Up
Source: Biomolecules. 2021 Jun 25;11(7):942. doi: 10.3390/biom11070942 (PMC8301975; doi:10.3390/biom11070942)
Supplement: Supplementary file 1 [file biomolecules-11-00942-s001.zip › biomolecules-1239596-supplementary.pdf]

Atelocollagen-embedded chondrocyte precursors as a treatment for high-grade cartilage defects of the femoral condyle: a case series with up to 9-year follow-up

Hwa-Chang Liu [1,2], Tzu-Shang Thomas Liu [3], Yen-Liang Liu [4], Jyh-Horng Wang [2], Chih-Hung Chang [5], Tiffany Ting-Fang Shih [6], and Feng-Huei Lin [7,*]

[1]Department of Orthopaedic Surgery, Taiwan Adventist Hospital, Taipei, Taiwan
[2]Department of Orthopaedic Surgery, National Taiwan University Hospital, Taipei, Taiwan
[3]Southern California Bone and Joint Clinic, California, U.S.A.
[4]Master Program for Biomedical Engineering, China Medical University, Taichung, Taiwan
[5]Department of Orthopaedic Surgery, Far Eastern Memorial Hospital, New Taipei, Taiwan
[6]Department of Radiology, National Taiwan University Hospital, Taipei, Taiwan
[7]Department of Biomedical Engineering, National Taiwan University, Taipei, Taiwan

*__Corresponding Authors:__
Feng-Huei Lin, Ph.D.
Professor
Department of Biomedical Engineering
National Taiwan University
Address: No. 1, Sec. 4, Roosevelt Rd., Taipei 10617, Taiwan (R.O.C.)

E-mail: double@ntu.edu.tw

Keywords: osteoarthritis, osteonecrosis, cartilage regeneration, chondrocyte precursor, mesenchymal stem cell

Contents

Method S1 | Comparison of chondrogenic differentiation protocols 3

Table S1 | Recipes of chondrogenic media 4

Table S2 | Primer sequences for chondrogenic genes 4

Method S2 | SCID mouse model for cartilage regeneration 5

Figure S1 | Procedures of chondrogenic induction 6

Figure S2 | Histological analysis of chondrogenic-induced MSCs 7

Figure S3 | Arthroscopy examination before and after CP implantation 8

Figure S4 | Grading of knee osteoarthritis by Kellgren and Lawrence system 9

Figure S5 | q-PCR gene analysis of MSCs during chondrogenic differentiation 10

Figure S6 | Histological analysis of extracellular matrix of the chondrogenic MSCs 14

Figure S7 | SCID mouse model for evaluating cartilage regeneration 15

Figure S8 | Histological analysis of implants in SCID mice (H&E, Alcian blue) 16

Figure S9 | Immunohistochemistry analysis of implants in SCID mice 17

Method S1 | Comparison of chondrogenic differentiation protocols

To compare the chondrocyte precursors (CPs) with conventional chondrogenic-induced mesenchymal stem cells (MSCs), we conducted histological analyses and mRNA gene expression profiling on the chondrogenic neotissues that were respectively prepared based on Nimni's[1] and our chondrogenic differentiation protocols as shown in **Table S1**. In Nimni's protocol, α-MEM with 0.35mg/ml atelocollagen was neutralized and mixed with human bone marrow-derived MSCs (cell density: 2×10^6 cells/150 μl). In Liu's protocol, the DMEM with 3 mg/ml atelocollagen was also neutralized and mixed with MSCs (cell density: 1×10^6 cells/ ml). The mixture of MSCs and atelocollagen was incubated in 37°C incubator in 15 mL centrifuge tube, and then the differentiation medium was added into tubes. The chondrogenic medium was changed every three days in Liu's protocol, and in Nimni's protocol in which four kinds of media were used during the chondrogenic differentiation. The schematic diagram of the chondrogenic procedure is shown in **Figure S1A**.

During the chondrogenic differentiation, the neotissues were examined by histological analysis (H&E staining images shown in **Figure S2**) and mRNA gene expression profiling (qPCR results are shown in **Figure S5**) on Day 0, 3, 7, 10, 14, and 21. The primer sequences of qPCR are listed in **Table S2.**

Liu's protocol					
Chondrogenic medium	DMEM				
	10 ng/ml hTGF-β1				
	ITS + Premix				
	50 ng/ml L-ascorbic acid-2-phosphate				
	10^{-7}M Dexamethasone				
**Nimni's protocol**					
Isolation medium	αMEM	0.5% FBS	1 ng/ml	hTGF-β1	
Expansion medium	αMEM	10% FBS	1 ng/ml	hTGF-β1	
Induction medium	αMEM	10% FBS	1 ng/ml	hTGF-β1	10^{-8}M Dexamethasone<br>2 mM B-Glycerol phosphate
Maintaining medium	αMEM	10% FBS	1 ng/ml	hTGF-β1	

Table S1 | Recipes of chondrogenic media

Primer		Accession no.	Forward 5'--3'	Reverse 5'--3'
1. Collagen I A1	COL1A1	NM_000088.3	5'-CAGCCGCTTCACCTACAGC-3'	5'-TTTTGTATTCAATCACTGTCTTGCC-3'
2. Collagen II A1	COL2A1	NM_001844.4	5'-CTGCAAAATAAAATCTCGGTGTTCT-3'	5'-GGGCATTTGACTCACACCAGT-3'
3. Collagen X A1	COL10A1	NM_000493.3	5'-TACCTTGTGCCTCCCATTCAA-3'	5'-TACAGTACAGTGCATAAATAAATAATATATCTCCA-3'
4. Aggrecan	ACAN	NM_013227.3	5'-GCCTCGGATCACCTGCAC-3'	5'-TGCCGTGAGCTCCGCT
5. MMP-3	MMP3	NM_002422.3	5'-AGACTTTCCAGGGATTGACTC-3'	5'-ACAATTAAGCCAGCTGTTACTCTT-3'
6. MMP-13	MMP13	NM_002427.3	5'-AGGCTCCGAGAAATGCAGTC-3'	5'-CCCCGCATCTTGGCTTTTTC-3'
7. Runx-2	RUNX2	NM_001015051.3	5'-CCCGTGGCCTTCAAGGT-3'	5'-CGTTACCCGCCATGACAGTA-3'
8. SOX-9	SOX9	NM_000346.3	5'-CTTTGGTTTGTGTTCGTGTTTTG-3'	5'-AGAGAAAGAAAAAGGGAAAGGTAAGTTT-3'
9. GAPDH	GAPDH	NM_002046.4	5'-CACTCAGACCCCCACCACAC-3'	5'-GATACATGACAAGGTGCGGCT-3'

Table S2 | Primer sequences for chondrogenic genes

Method S2 | SCID mouse model for cartilage regeneration

To compare the CP therapy with mosaicplasty, we developed a cartilage regeneration model using severe combined immunodeficient (SCID) mice. Human cartilage tissues were acquired from patients who received total knee replacement. The cartilage tissues were trimmed into a disc-like shape with a diameter of 10 mm and a thickness of 2 mm. The center of the cartilage disc was punched using a biopsy punch with a diameter of 3 mm. In the group with mosaicplasty treatment, the punched cartilage was inserted back to the cartilage disc. In the group with CP therapy, the hole was filled with CPs. These constructs were later subcutaneously implanted into SCID mice. The mice were sacrificed after 6 months, and the implants were examined using histological analyses and real-time PCR to evaluate the cartilage regeneration (**Figure S7**). The implants were carefully cut into half to create paraffin blocks. The cross-sections of implants were investigated using H&E staining (**Figure S8A**), Alcian blue staining (**Figure S8B**), and immunohistochemistry (**Figure S9**). The flowchart of the SCID mouse model is listed below:

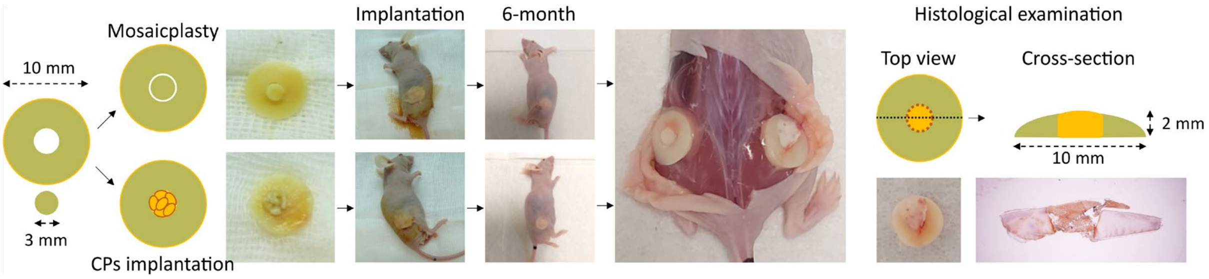

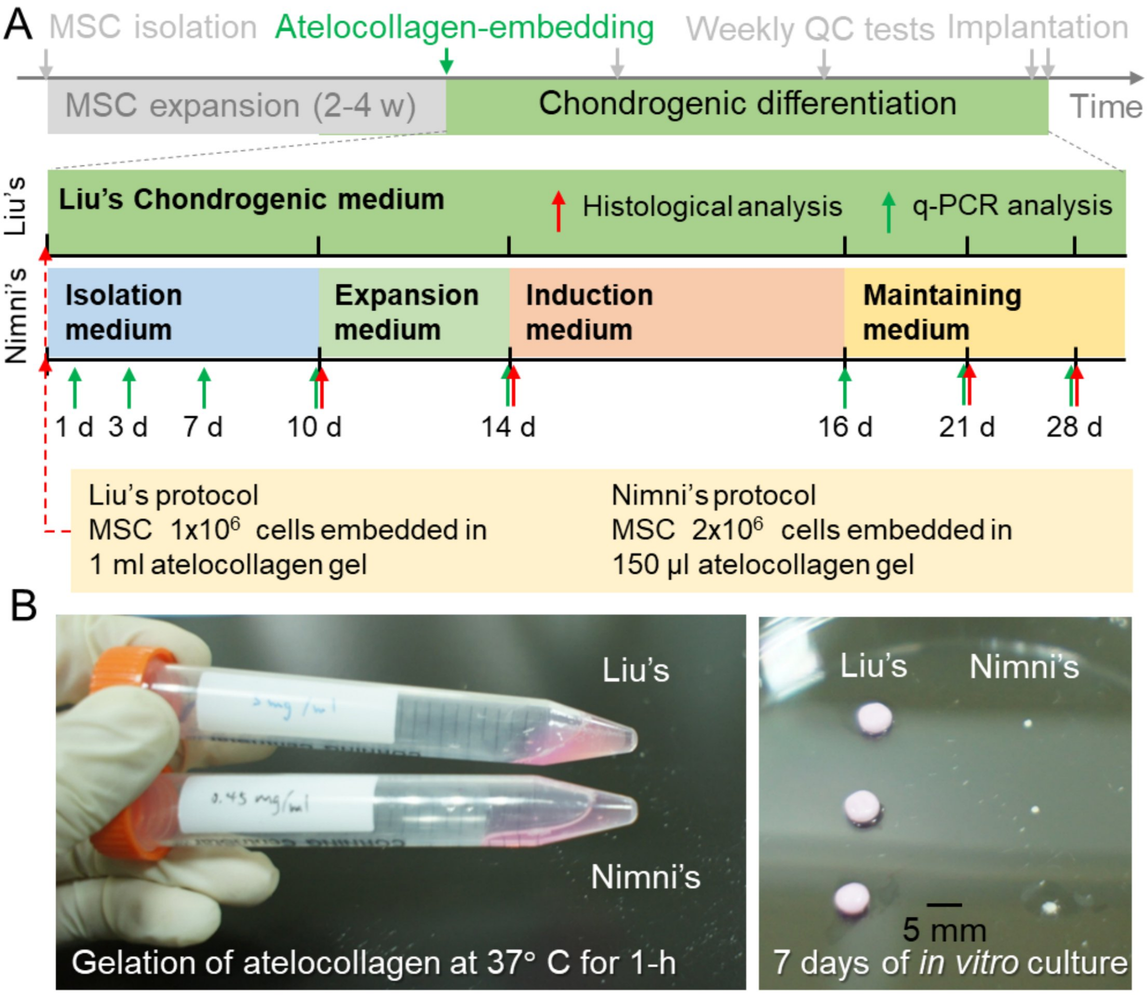

Figure S1 | Procedures of chondrogenic induction

(**A**) Procedures of chondrogenic induction. To investigate the development of the chondrogenic neotissues under different induction conditions, the morphogenesis and gene expression were analyzed using H&E staining and q-PCR at different time points (red arrows for histological analysis and green arrows for q-PCR) during the chondrogenic differentiation. (**B**) The MSC-atelocollagen mixture can form a gel within 1 hour under 37°C using Liu's protocol, and chondrogenic neotissues prepared in Liu's procedure were significantly larger than those prepared in Nimni's procedure after 7 days of *in vitro* culture.

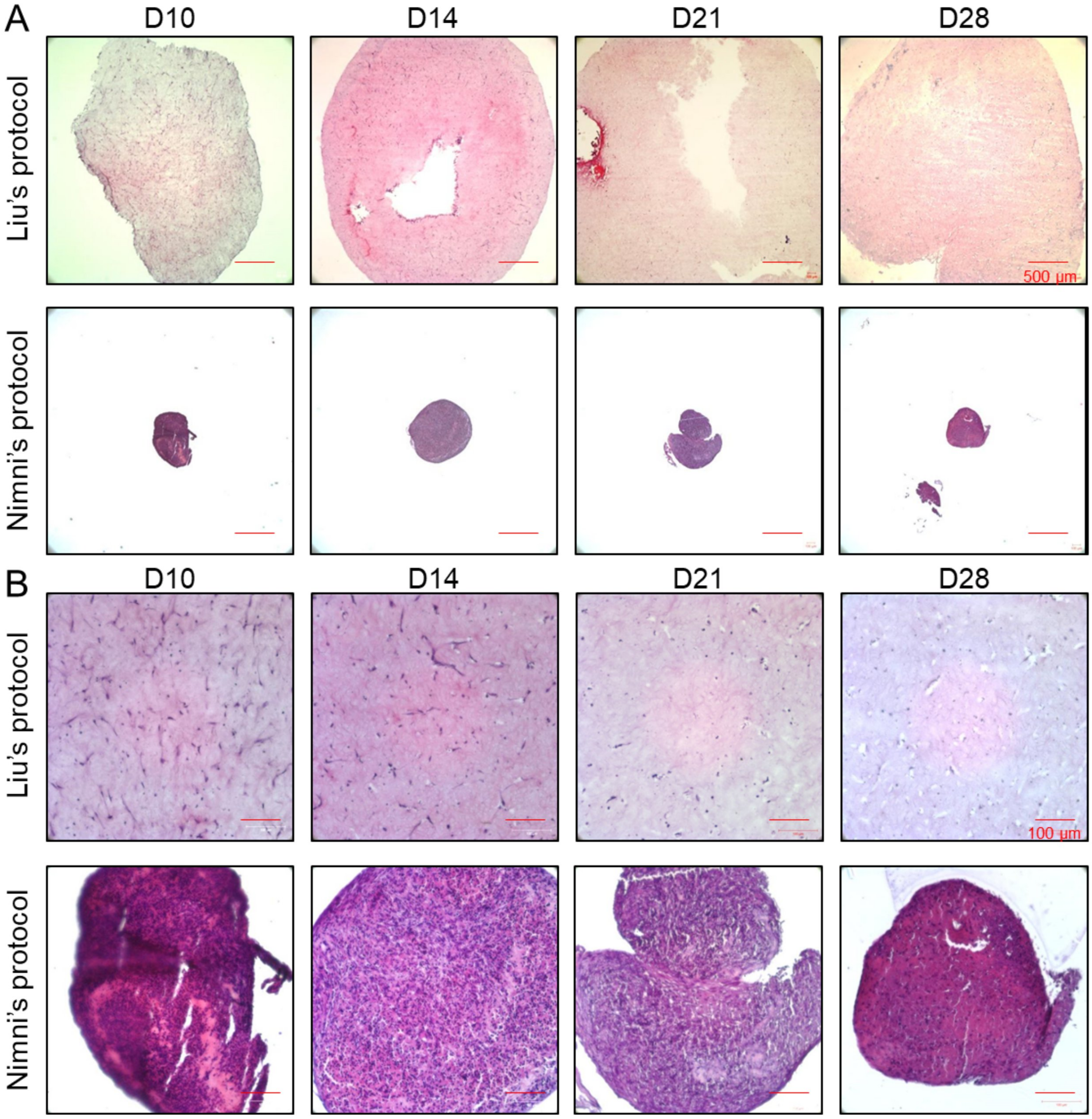

Figure S2 | Histological analysis of chondrogenic-induced MSCs

(**A**) Histological images of chondrogenic neotissues. Liu's protocol created neotissues with a diameter of ~3 mm, and Nimni's protocol generated neotissues with a diameter of ~0.5 mm. (**B**) The histological images with a higher magnification.

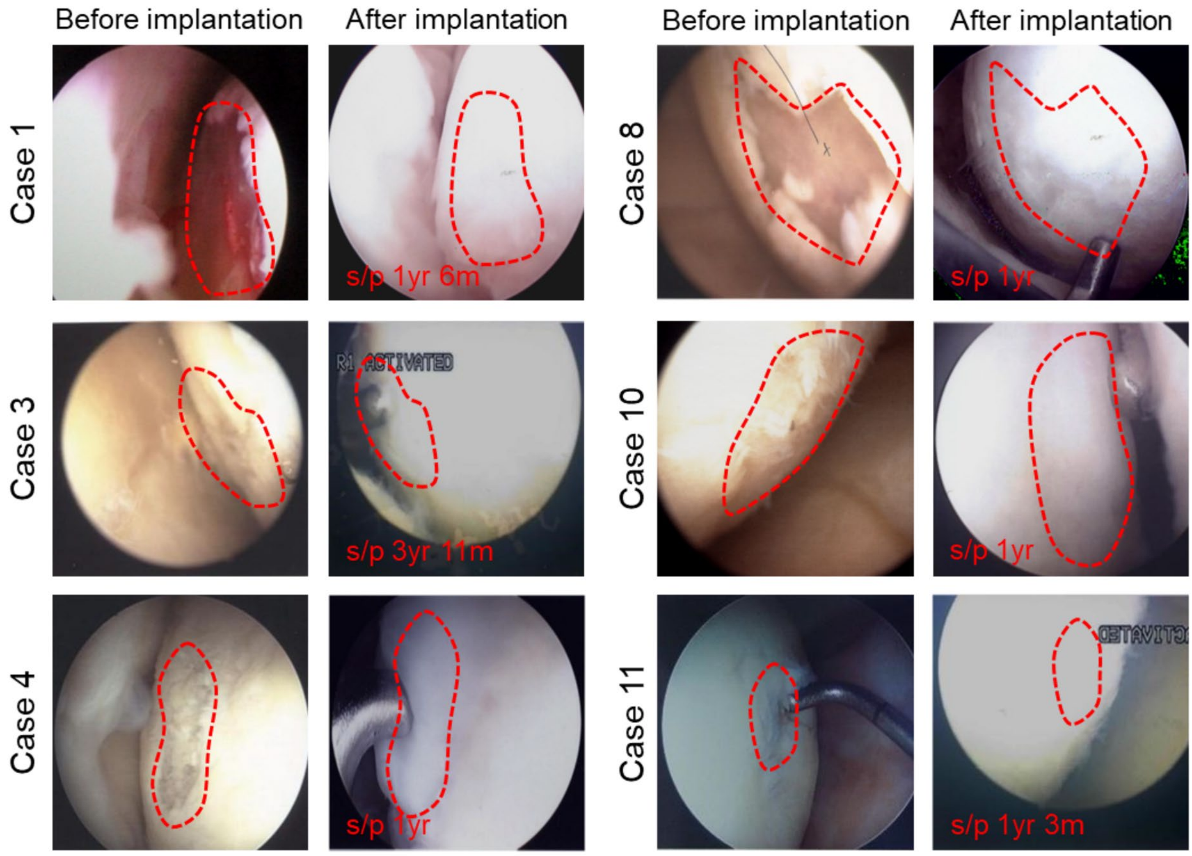

Figure S3 | Arthroscopy examination before and after CP implantation

The defect sites are circled by the red dash lines in the arthroscopy images and indicated by the red arrows on the other three types of images.

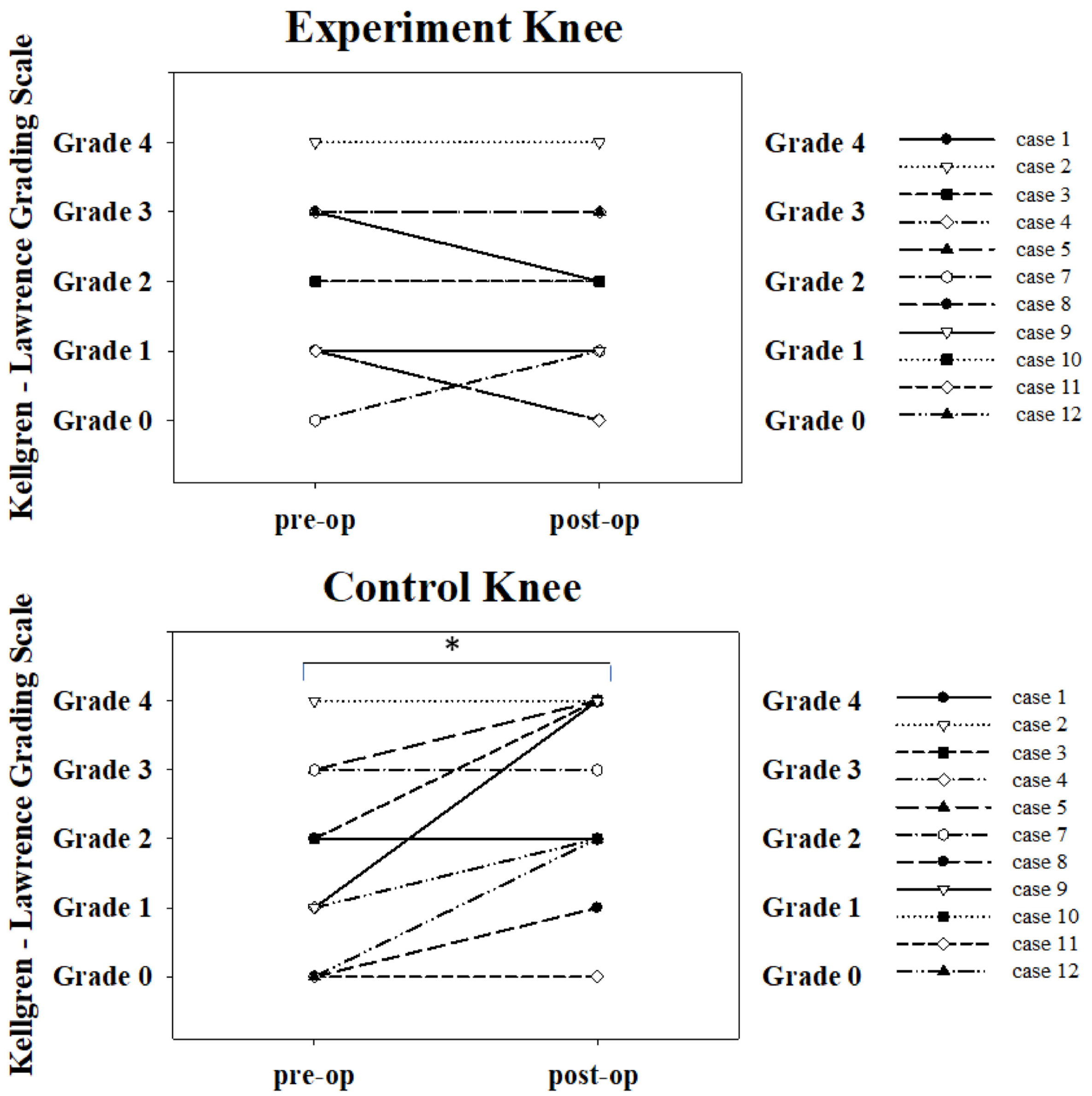

Figure S4 | Grading of knee osteoarthritis by Kellgren and Lawrence system

The CP-implanted knees (experimental group) also showed no substantial changes in K-L grade (-0.19 grade in average with $p = 0.34$), whereas untreated contralateral knees (control group) developed severer OA (+0.90 grade with $p = 0.016$) one year after CP therapy. The grading of K-L system: Grade 0 - no radiographic features of OA are present; Grade 1 - doubtful joint space narrowing (JSN) and possible osteophytic lipping; Grade 2 - the presence of definite osteophytes and possible JSN on anteroposterior weight-bearing radiograph; Grade 3 - multiple osteophytes, definite JSN, sclerosis, possible bony deformity; Grade 4 - large osteophytes, marked JSN, severe sclerosis and definitely bony deformity.

Figure S5 | q-PCR gene analysis of MSCs during chondrogenic differentiation

In both protocols, the COL2A1 and ACAN expressions were upregulated; however, the neotissues prepared using Liu's procedure exhibited gradual increases rather than the abrupt increases at day 7 followed by decreases along time in Nimni's procedure. The similar expression patterns were also shown in the expression of MMP-3 and MMP-13. We hypothesize that gradual increases of cartilage-regeneration-related genes would be beneficial for both cartilage maturation and integration of implanted neotissues and original cartilage.

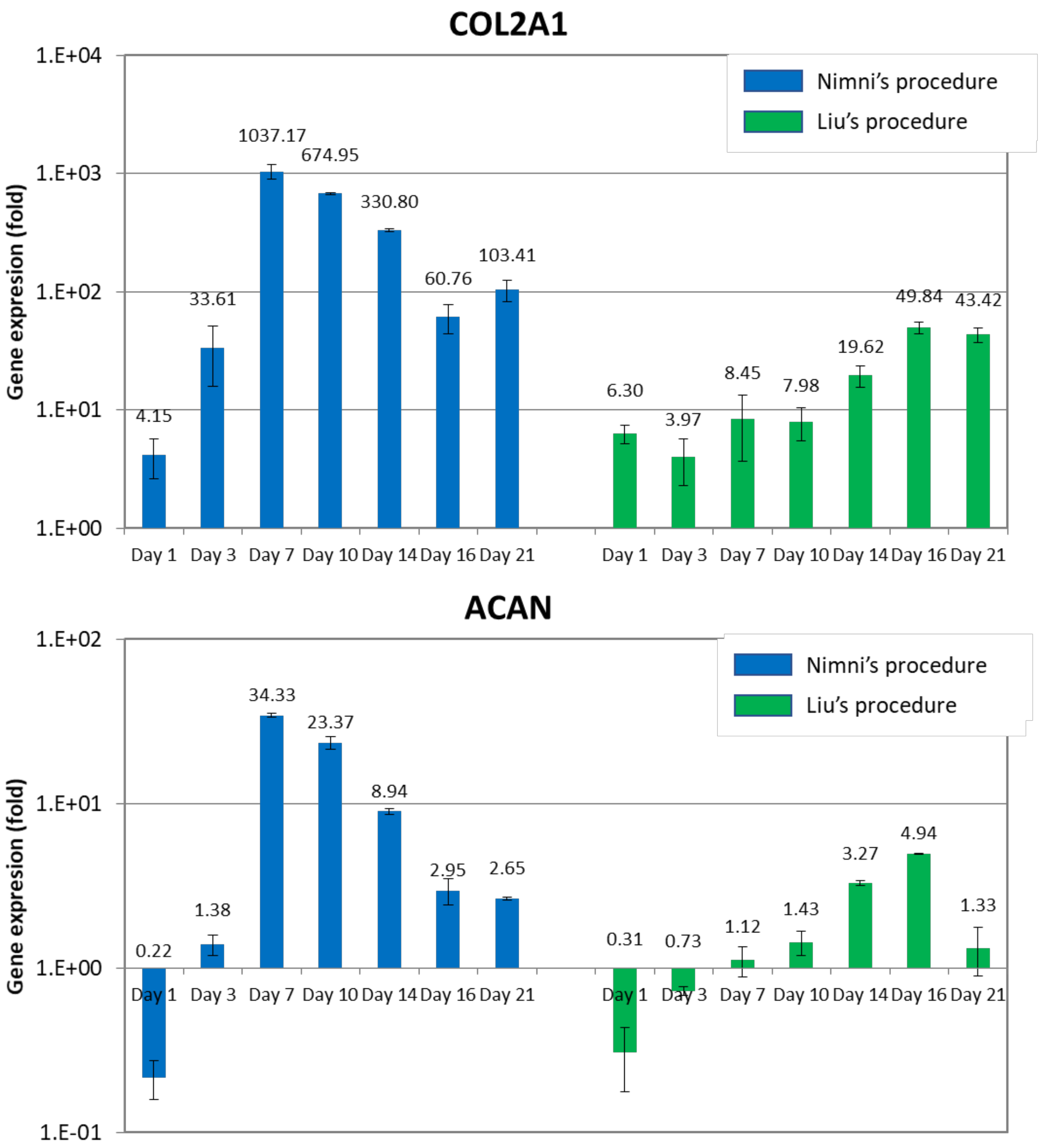

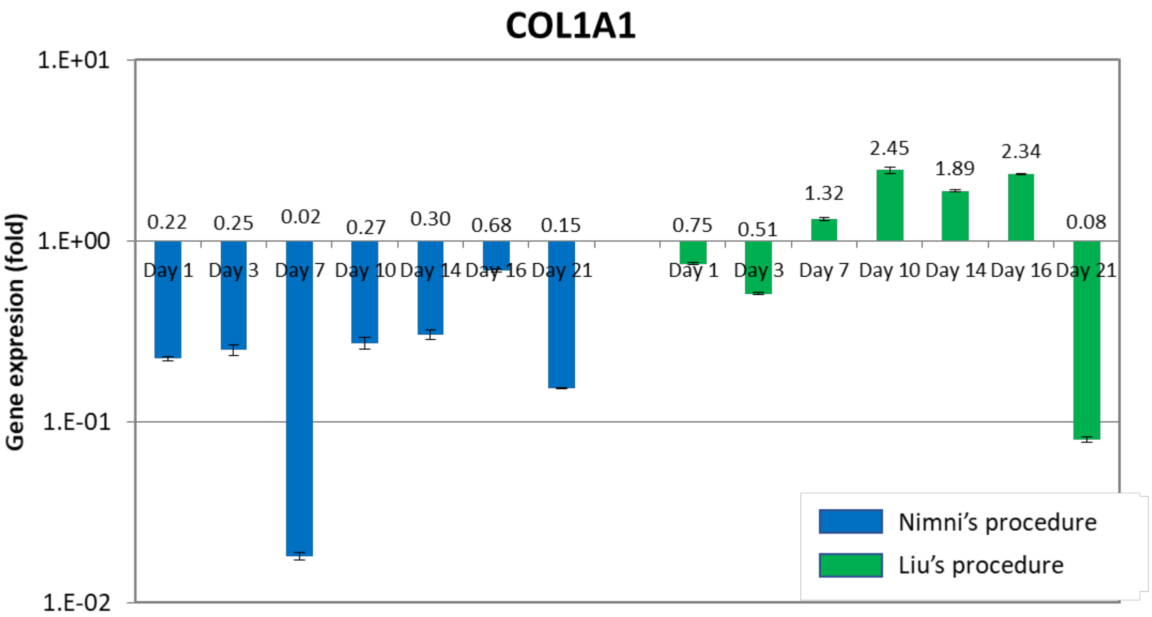
COL1A1
1.E+01
1.E+00
1.E-01
1.E-02
Gene expresion (fold)
0.22
0.25
0.02
0.27
0.30
0.68
0.15
0.75
0.51
1.32
2.45
1.89
2.34
0.08
Day 1
Day 3
Day 7
Day 10
Day 14
Day 16
Day 21
Day 1
Day 3
Day 7
Day 10
Day 14
Day 16
Day 21
Nimni's procedure
Liu's procedure

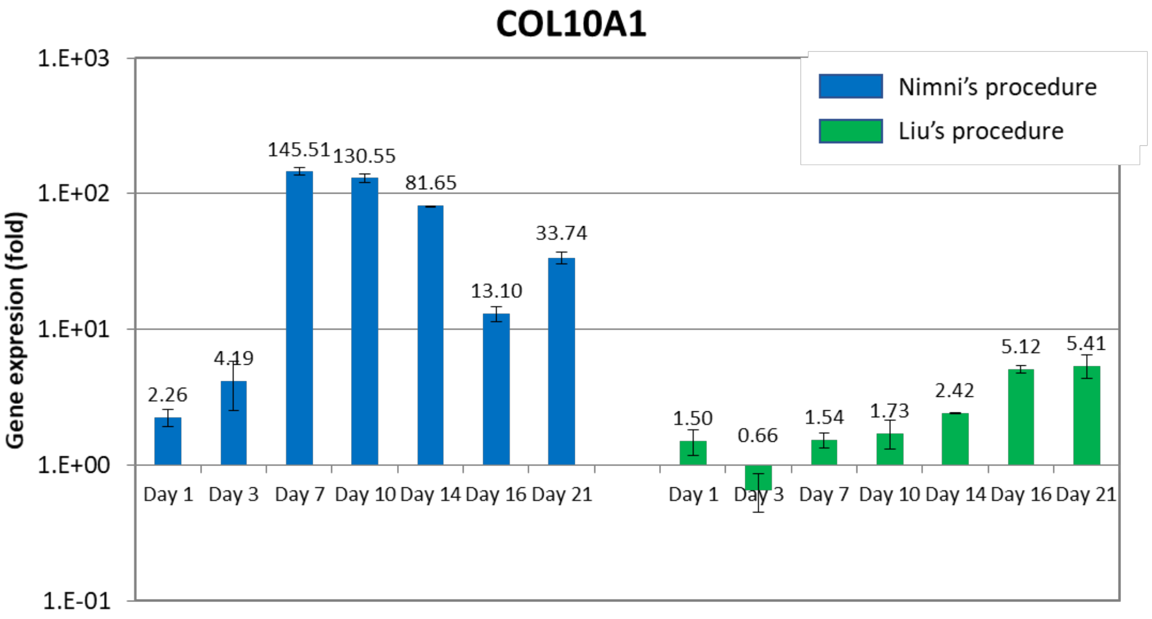
COL10A1
1.E+03
1.E+02
1.E+01
1.E+00
1.E-01
Gene expresion (fold)
Nimni's procedure
Liu's procedure
2.26
4.19
145.51
130.55
81.65
13.10
33.74
1.50
0.66
1.54
1.73
2.42
5.12
5.41
Day 1
Day 3
Day 7
Day 10
Day 14
Day 16
Day 21
Day 1
Day 3
Day 7
Day 10
Day 14
Day 16
Day 21

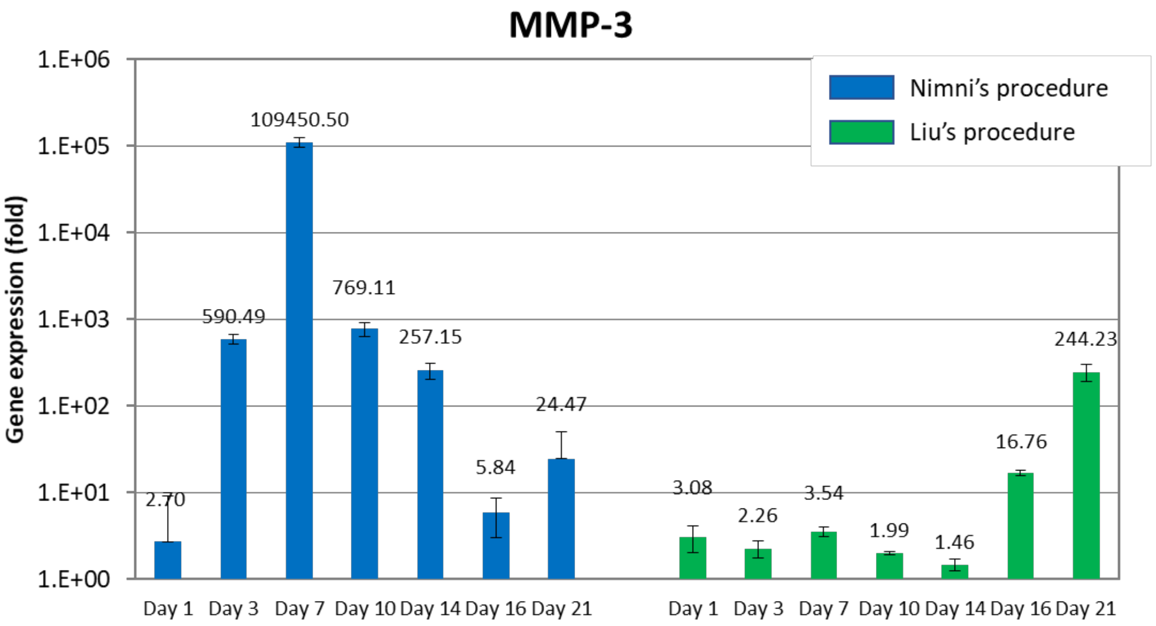
MMP-3
Nimni's procedure
Liu's procedure
Gene expression (fold)
1.E+06
1.E+05
1.E+04
1.E+03
1.E+02
1.E+01
1.E+00
2.70
590.49
109450.50
769.11
257.15
5.84
24.47
3.08
2.26
3.54
1.99
1.46
16.76
244.23
Day 1 Day 3 Day 7 Day 10 Day 14 Day 16 Day 21
Day 1 Day 3 Day 7 Day 10 Day 14 Day 16 Day 21

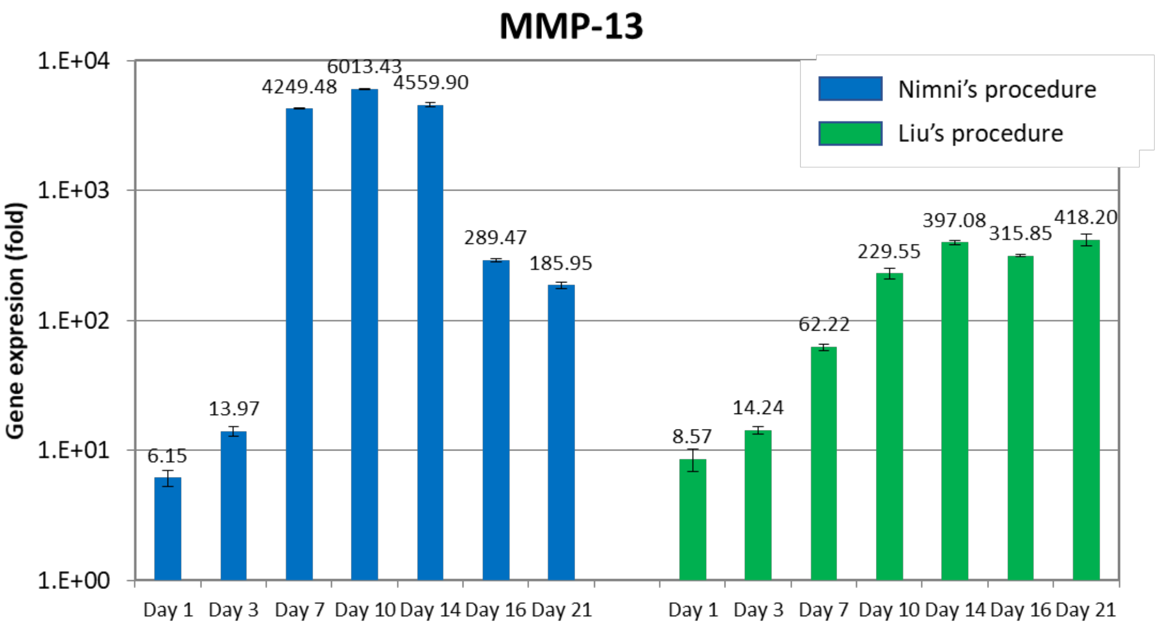
MMP-13
Nimni's procedure
Liu's procedure
Gene expresion (fold)
1.E+04
1.E+03
1.E+02
1.E+01
1.E+00
6.15
13.97
4249.48
6013.43
4559.90
289.47
185.95
8.57
14.24
62.22
229.55
397.08
315.85
418.20
Day 1 Day 3 Day 7 Day 10 Day 14 Day 16 Day 21
Day 1 Day 3 Day 7 Day 10 Day 14 Day 16 Day 21

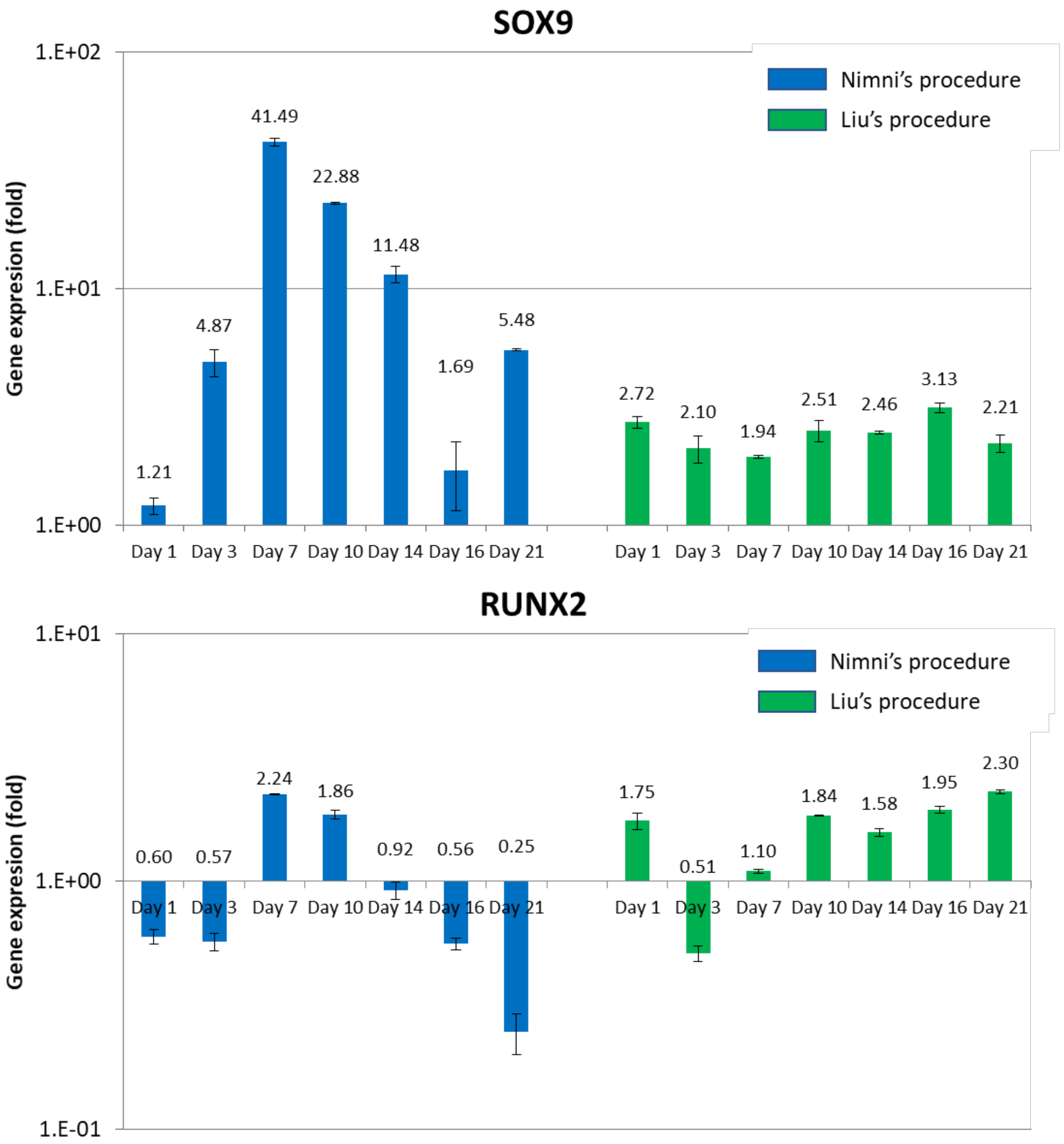

SOX9
Nimni's procedure
Liu's procedure
Gene expresion (fold)
1.E+02
1.E+01
1.E+00
41.49
22.88
11.48
4.87
5.48
1.69
1.21
2.72
2.10
1.94
2.51
2.46
3.13
2.21
Day 1 Day 3 Day 7 Day 10 Day 14 Day 16 Day 21
Day 1 Day 3 Day 7 Day 10 Day 14 Day 16 Day 21
RUNX2
Nimni's procedure
Liu's procedure
Gene expresion (fold)
1.E+01
1.E+00
1.E-01
0.60
0.57
2.24
1.86
0.92
0.56
0.25
1.75
0.51
1.10
1.84
1.58
1.95
2.30
Day 1 Day 3 Day 7 Day 10 Day 14 Day 16 Day 21
Day 1 Day 3 Day 7 Day 10 Day 14 Day 16 Day 21

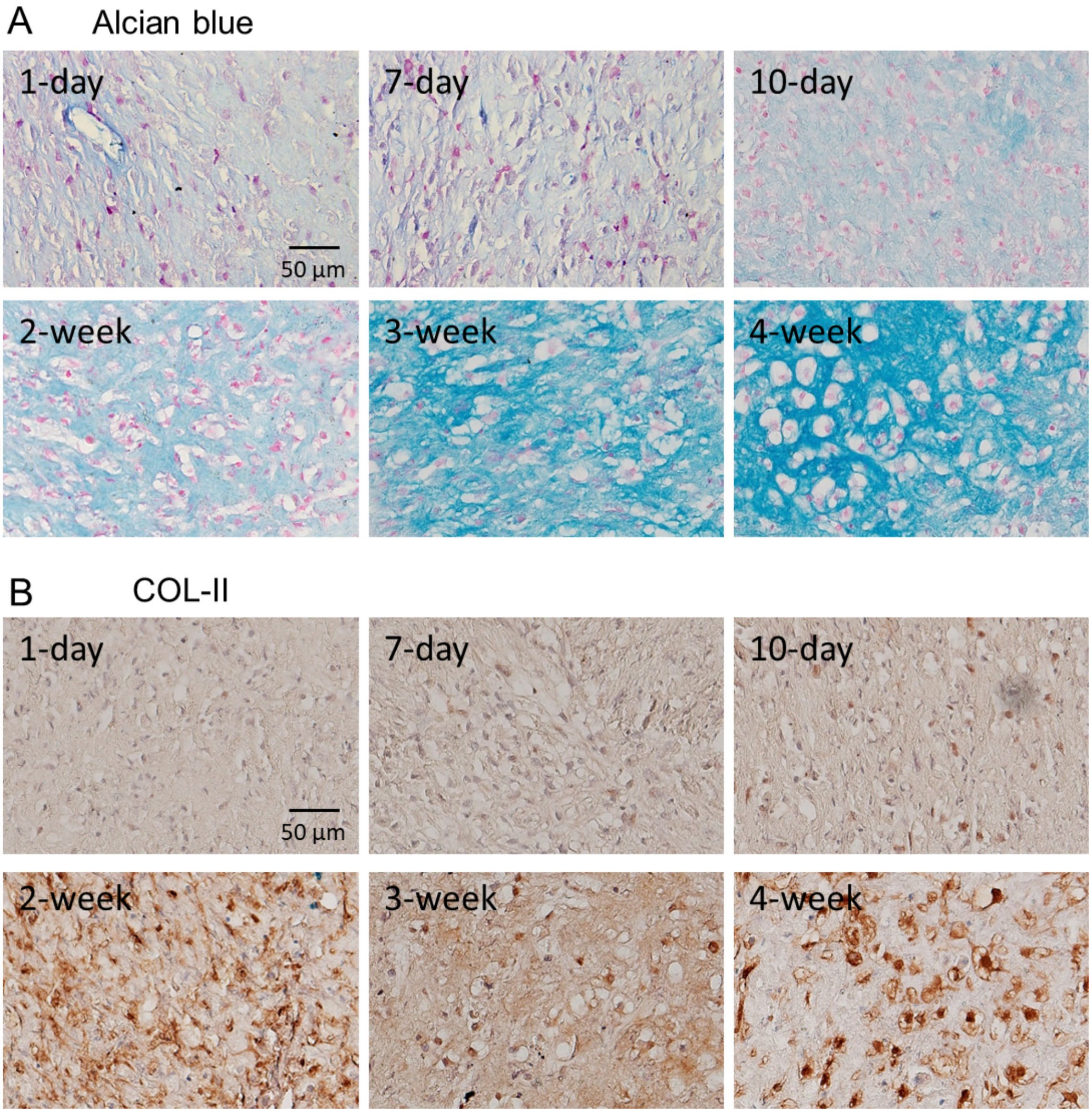

Figure S6 | Histological analysis of extracellular matrix of the chondrogenic MSCs

Alcian blue staining and immunohistochemistry were performed to visualize the tissue morphology and expression levels of GAG (A) and collagen type II (COL-II) (B), respectively.

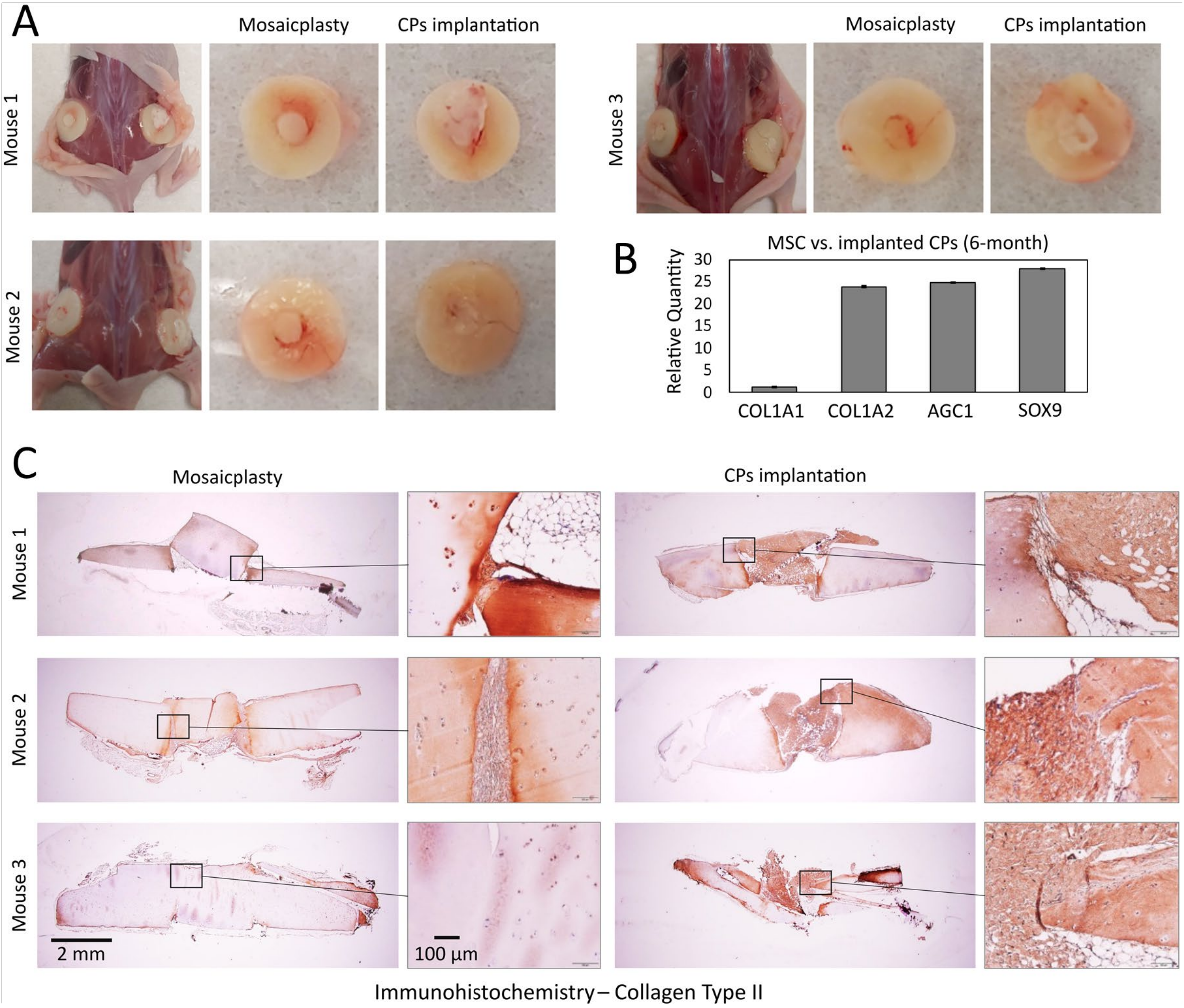

Figure S7 | SCID mouse model for evaluating cartilage regeneration

(A) Appearance of implanted cartilage with the treatments either mosaicplasty or CPs implantation. (B) Relative mRNA expressions of COLA1, COLA2, AGC1, and SOX9 by comparing mesenchymal stem cells and the implanted CPs. (C) Histological sections of the implanted cartilages. Immunohistochemistry was performed to visualize the tissue morphology and expression levels of collagen type II.

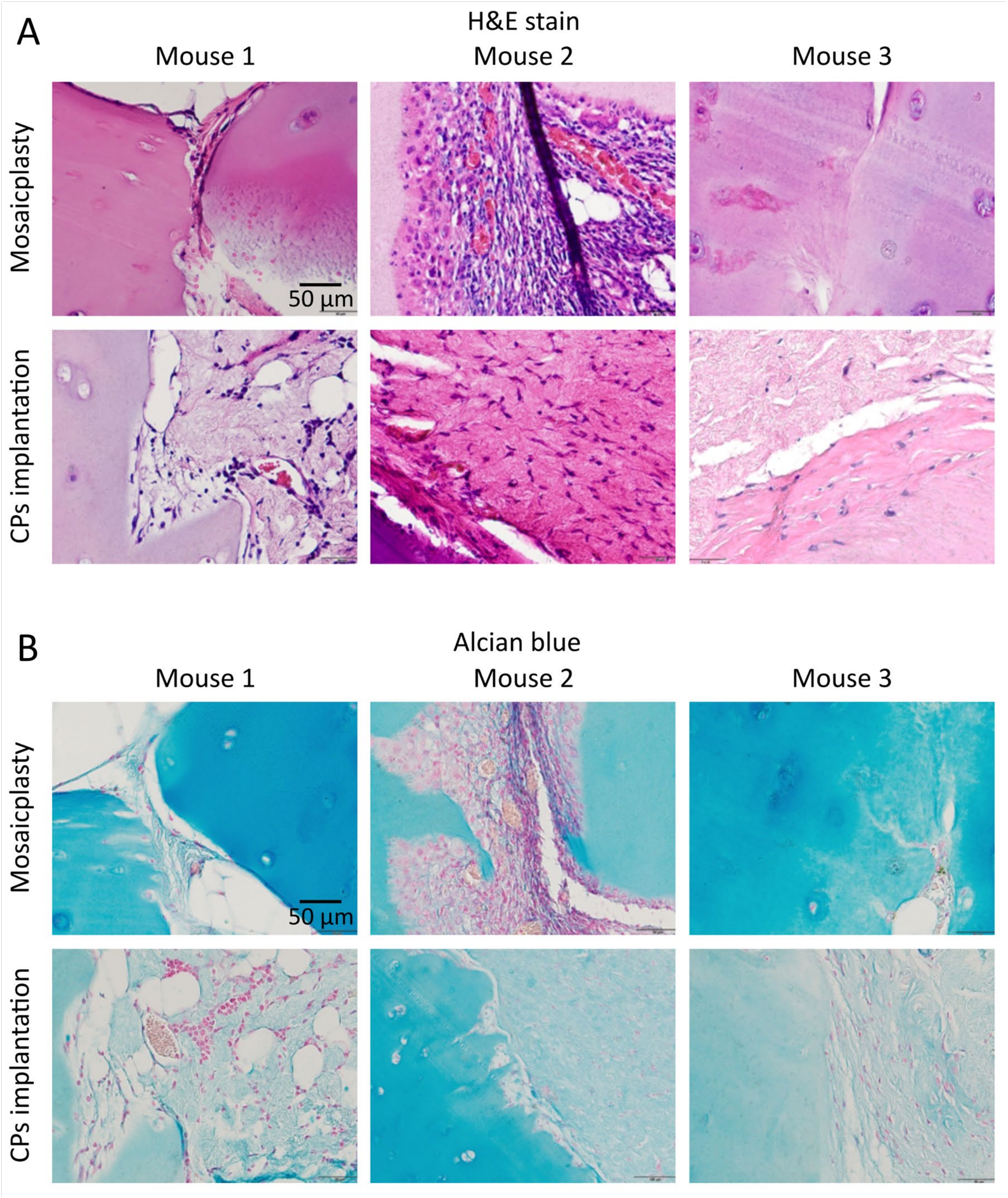

Figure S8 | Histological analysis of implants in SCID mice (H&E, Alcian blue)

H&E staining and Alcian blue staining were performed to visualize the tissue morphology and expression levels of GAG (B).

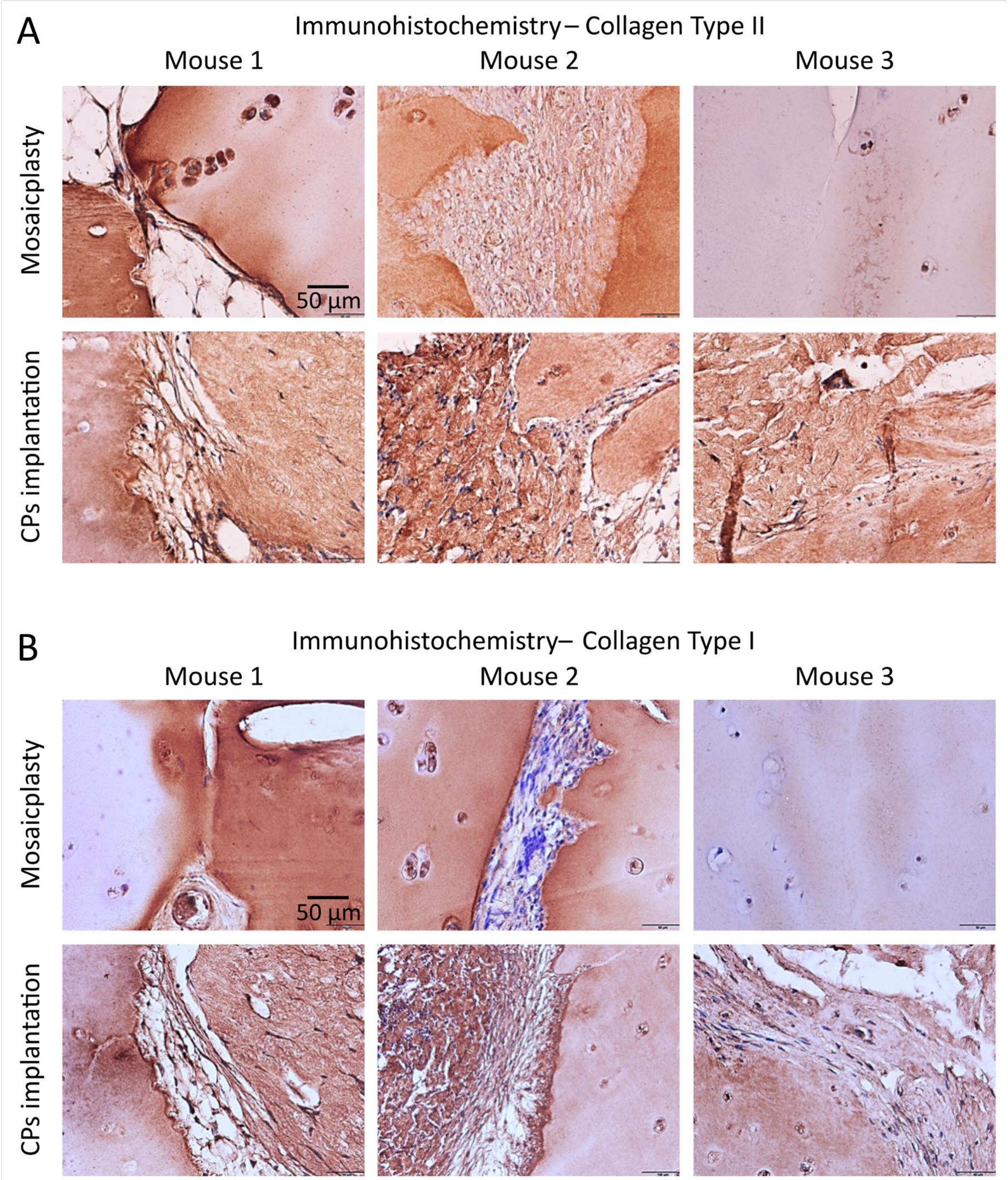

Figure S9 | Immunohistochemistry analysis of implants in SCID mice

Immunohistochemistry was performed to visualize the expression levels of collagen type II (A) and collagen type I (B), respectively.

REFERENCE:

1. Andrades JA, Han B, Becerra J, Sorgente N, Hall FL, Nimni ME. A recombinant human TGF-β1 fusion protein with collagen-binding domain promotes migration, growth, and differentiation of bone marrow mesenchymal cells. Exp Cell Res 1999;250:485-98.
